# Supplementary material for: Analysis of aPTT predictors after unfractionated heparin administration in intensive care units using machine learning models
Source: PLoS One. 2025 Jul 21;20(7):e0328709. doi: 10.1371/journal.pone.0328709 (PMC12279130; doi:10.1371/journal.pone.0328709)
Supplement: S1 File — S1 Fig: A Venn diagram illustrating the number of data points used to construct and evaluate each prediction model. S2 Fig: Beeswarm diagrams showing contributing variables to predictive performance in both first and multiple heparin infusion models. (DOCX) [file pone.0328709.s001.docx]

**
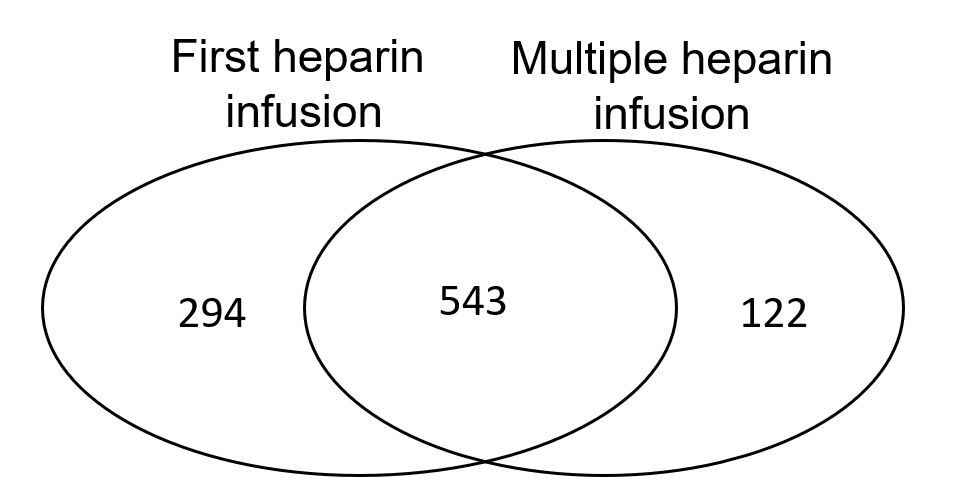
****Supplementary Figure S1**

Venn diagram illustrating the number of data points used to construct and evaluate each prediction model for first and multiple heparin infusions, based on a dataset of 959 eligible cases.

An example of the 294 data points exclusive to the “first heparin infusion” category is when heparin medication was discontinued (see Supplementary Table S2, Case 3, for details). Similarly, an example of the 122 data points specific to “multiple heparin infusions” is when the target aPTT was not measured (see Supplementary Table S2, Case 2, for details).

**Supplementary Figure S2**


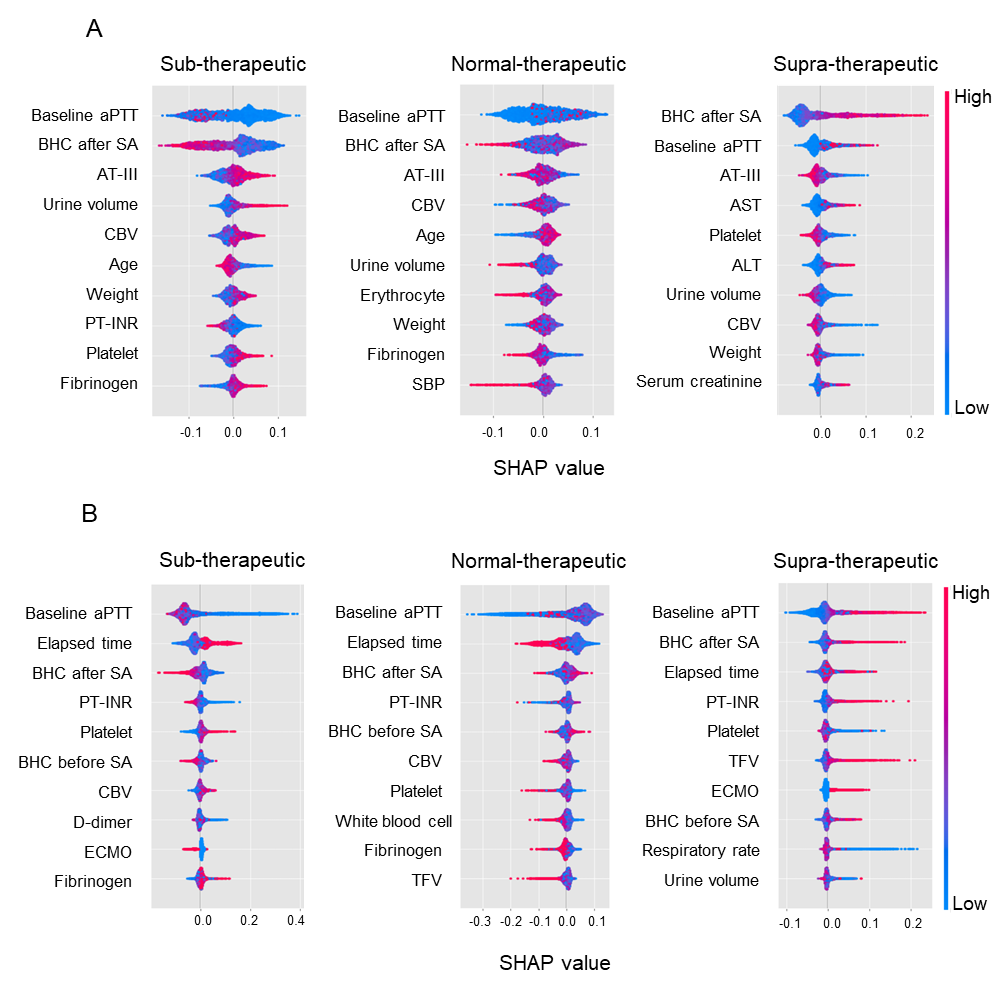
Contributing variables to predictive performance in (A) first and (B) multiple heparin infusion patients, as represented in the beeswarm diagram.

The contributions to the prediction model were determined using a random forest model, with data imputed based on static variables, for both first and multiple heparin infusion patient predictions. aPTT: activated partial thromboplastin time; BHC after SA: blood heparin concentration after the starting point of administration; AT-III: antithrombin III; Urine volume: amount of urine output; CBV: circulation blood volume; PT-INR: prothrombin time-international normalized ratio; SBP: systolic blood pressure; AST: aspartate aminotransferase; ALT: alanine aminotransferase; Elapsed time: elapsed time from baseline aPTT measurement to target aPTT measurement; BHC before SA: blood heparin concentration before the starting point of administration; ECMO: extracorporeal membrane oxygenation; TFV: total fluid volume.
