# Supplementary material for: Analysis of aPTT predictors after unfractionated heparin administration in intensive care units using machine learning models
Source: PLoS One. 2025 Jul 21;20(7):e0328709. doi: 10.1371/journal.pone.0328709 (PMC12279130; doi:10.1371/journal.pone.0328709)
Supplement: S2 File — S1 Table: Exclusions in the data processing and model construction procedure. S2 Table: Hyperparameter tuning settings for each machine learning model used in the study. S3 Table: Confusion matrix and calculation methods for evaluation metrics. (DOCX) [file pone.0328709.s002.docx]

**Supplementary Table S1.**
Exclusions in the data processing and model construction procedure

| Case1. Baseline aPTT were not measured. | Detailed description: |
| --- | --- |
| 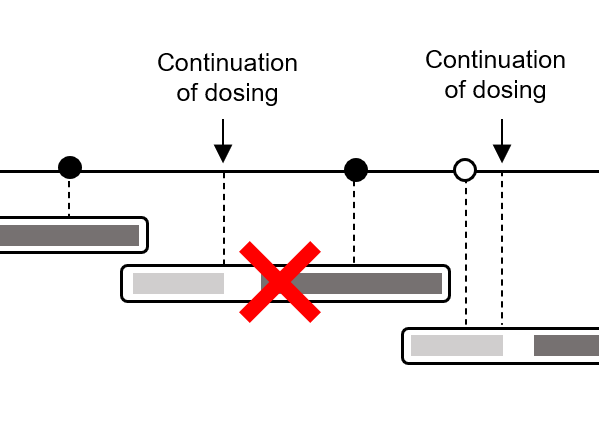 | No aPTT were recorded within 24 hours prior to time of heparin administration. |
| Case2. Target aPTT were not measured |  |
| 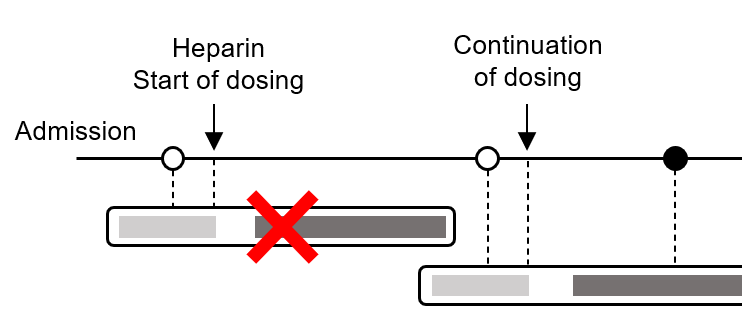 | No aPTT were recorded in the range of 6 to 24 hours after the time of heparin dosing |
| Case3. Heparin medication was discontinued |  |
| 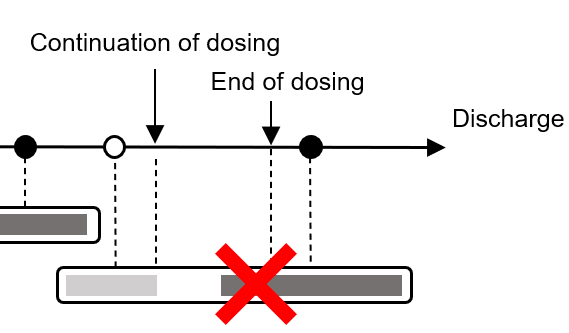 | Heparin dosing completed by target aPTT from time of heparin administration. |
| Case4. Heparin medication dosage was changed. |  |
| 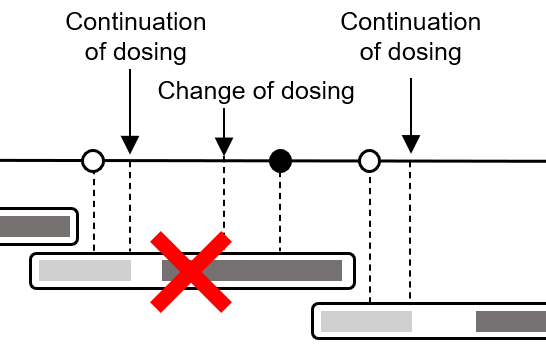 | Dosing rate changes from time of heparin dosing to target aPTT |

**Supplementary Table S2.**
A. Hyper parameter tuning of each model

| Model name | Hyper parameter |
| --- | --- |
| Logistic regression | random_state = 0  multi_class = "ovr"  *The others = default |
| SVC | random_state = 0  *The others = default |
| XGB | random_state = 0  objective = "multi:softprob"  eval_metric = ["merror","mlogloss"]  tree_method = "gpu_hist"  * The others = default |
| Random forest | random_state = 0  * The others = default |
| LightGBM | random_state = 0  metric = "multi_logloss"  objective = "multiclass"  * The others = default |
| Multineural network | Learning rate = 0.0001  Dropout = 0.5  Batch size = 16  <Static>  Filter of vitals = [16, 18, 24]  Filter of output = [2, 8, 16]  Number of nodes in the first fully connected layer = [32, 48]  Number of nodes in the second fully connected layer = [16, 32]  <Time-series & static>  Filter of vitals = [16, 18, 24]  Filter of output = [2, 8, 16]  Number of nodes in the first fully connected layer = [32, 48]  Number of nodes in the second fully connected layer = [16, 32]  *The final hyperparameters are indicated in bold. |

B. Packages and modules used for each model

| Model name | Package name | Module name |
| --- | --- | --- |
| Logistic regression | sklearn.linear_model | LogisticRegression |
| SVC | sklearn.svm | SVC |
| XGB | xgboost.sklearn | XGBClassifier |
| Random forest | sklearn.ensemble | RandomForest Classifier |
| LightGBM | lightgbm | LGBMClassifier |

**Supplementary Table S3.**

Confusion matrix (A) and calculation method of evaluation index (B)

A. Confusion matrix

|  |  | Predicted | | |
| --- | --- | --- | --- | --- |
|  |  | sub-therapeutic | normal-therapeutic | supra-therapeutic |
|  | sub-therapeutic | A | B | C |
| Actual | normal-therapeutic | D | E | F |
|  | supra-therapeutic | G | H | I |

B. Calculation method of evaluation index

| Precision | Sub-therapeutic | A / (A＋D＋G) … P_x |
| --- | --- | --- |
|  | Normal-therapeutic | E / (B＋E＋H) … P_y |
|  | Supra-therapeutic | I / (C＋F＋I) … P_z |
|  | Macro | (P_x + P_y + P_z ) / 3 |
| Recall | Sub-therapeutic | A / ( A + B + C ) … R_x |
|  | Normal-therapeutic | E / ( D＋E＋F ) … R_y |
|  | Supra-therapeutic | I / (G＋H＋I) … R_z |
|  | Macro | (R_x + R_y + R_z ) / 3 |
| F1 | Sub-therapeutic | 2 * P_x * R_x / (P_x + R_x) … F_x |
|  | Normal-therapeutic | 2 * P_y * R_y / (P_y + R_y) … F_y |
|  | Supra-therapeutic | 2 * P_z * R_z / (P_z + R_z) … F_z |
|  | Macro | (F_x + F_y + F_z ) / 3 |
