# Supplementary material for: Analysis of aPTT predictors after unfractionated heparin administration in intensive care units using machine learning models
Source: PLoS One. 2025 Jul 21;20(7):e0328709. doi: 10.1371/journal.pone.0328709 (PMC12279130; doi:10.1371/journal.pone.0328709)
Supplement: Attachment — Submitted filename: Paper Review -PLOS.docx [file pone.0328709.s004.docx]

The authors proposed a method to predict aPTT using Machine Learning. In general, the paper is well structured and easy to read. The results are well explained and the method is presented in an organized and easy to read structure.

I recommend the authors to do minor modifications before publishing this work as follows:

1- Introduction:
   a. Since there is no dedicated section for previous work, the introduction should cover insights about the gap and what others have done in this domain. The introduction is very short and lacks the depth of analysis on published research. You need to discuss how is this study different from others. For example:
      i. Abdel-Hafez, Ahmad, Ian A. Scott, Nazanin Falconer, Stephen Canaris, Oscar Bonilla, Sven Marxen, Aaron Van Garderen, and Michael Barras. "Predicting therapeutic response to unfractionated heparin therapy: machine learning approach." *Interactive journal of medical research* 11, no. 2 (2022): e34533.
      ii. Ghassemi MM, Richter SE, Eche IM, Chen TW, Danziger J, Celi LA. A data-driven approach to optimized medication dosing: a focus on heparin. Intensive Care Med 2014 Sep 5;40(9):1332-1339
      iii. Lin R, Stanley MD, Ghassemi MM, Nemati S. A deep deterministic policy gradient approach to medication dosing and surveillance in the ICU. Annu Int Conf IEEE Eng Med Biol Soc 2018 Jul;2018:4927-4931
      iv. And Others
   b. Sentences line 80 and 82 require referencing
   c. Line 86: mention the dataset size explicitly
   d. Line 87: spell out the static and dynamic features
2- In Data Source can you add a table of dataset details from the 7 hospitals (show stats of your dataset)
3- In line 124, is there a time frame for when aPTT is conducted after the bolus dose to be included? (Noticed it is mentioned in line 190, it is better to mention this in inclusion/exclusion criteria)
4- In line 138 you defined sub-therapeutic (< 40 s), normal-therapeutic (40–80 s), and supra

therapeutic (> 80 s), is this consistent with other research ?? I noticed therapeutic range is defined as 70-100 in other work Can you justify this selection?

5- Since this is a multiclassification (3 categories) it is not clear to me how you calculated performance metrics (is it micro-precision or macro-precision?) can you provide precision and recall for every category (sub-therapeutic, normal-therapeutic, and supra-therapeutic).
