# Supplementary material for: Analysis of aPTT predictors after unfractionated heparin administration in intensive care units using machine learning models
Source: PLoS One. 2025 Jul 21;20(7):e0328709. doi: 10.1371/journal.pone.0328709 (PMC12279130; doi:10.1371/journal.pone.0328709)
Supplement: Attachment — Submitted filename: Reviewer_comment_response_auresp_2.docx [file pone.0328709.s006.docx]

**Reviewer #1:**

1. **The abstract is somewhat verbose. Quantitative results (e.g., AUC values) should be clearly highlighted in the results section. The conclusion is too strong and relatively inaccurate. 71%AUC is acceptable but don’t indicate good performance.**

Thank you for your valuable comments. We appreciate your insights and have revised the Results and Conclusions sections accordingly to improve clarity and accuracy.

We have addressed your concern regarding the overstatement of model performance by modifying the wording in the Results section to more accurately reflect the predictive accuracy of the models. Additionally, we have clarified that the AUC values indicate moderate predictive performance rather than suggesting strong performance. Furthermore, in the Conclusions section, we have refined our statements to avoid overgeneralization and have emphasized the need for further research to improve predictive accuracy. The revised sections are as follows:

Results: The ML models exhibited high accuracy in predicting aPTT following both initial and multiple heparin doses. The random forest model achieved the highest ROC AUC, with values of 0.71 for the initial dose and 0.73 for multiple doses. Key predictive factors across both models included the prior aPTT levels and heparin blood concentration.

Conclusions: The random forest model successfully predicted the aPTT response to unfractionated heparin in ICU patients. The inclusion of static and dynamic features did not enhance the prediction accuracy and no clear trend was observed in further improving the model performance. Future studies should explore additional factors to refine predictive models for optimizing individualized anticoagulation management in ICUs.

2. **While the introduction outlines the context well, it lacks a comprehensive review of prior studies. It would benefit from a clearer delineation of how this study builds upon or contrasts with existing literature.Explicitly state the research gap that this study addresses. This will clarify its relevance and importance.**

Thank you for your insightful comment. We agree with your suggestion and have revised the Introduction to include a discussion of existing studies, making the research gap more explicit and clearly highlighting how this study builds upon or contrasts with prior work.

3. **While the study involves a large cohort, a statistical rationale for the sample size should be included. How was the sample size determined? Were power analyses conducted?**

Thank you for your insightful comment. In response to your concern, we conducted post hoc sample size calculations for evaluating the ROC AUC using the pROC package in R. Assuming an ROC AUC of 0.7, a power of 0.90, and a one-tailed test, we determined that a minimum of 48 samples was required for the first-dose group and 94 samples for the multiple-dose group. As shown in Figure 3, this consideration was particularly important since the Supra-therapeutic group comprised approximately 20–30% of the other groups. In our study, we included 151 and 418 samples, respectively, confirming that a sufficient sample size was achieved.

4. **The authors mention using SHAP for model interpretation. A clearer explanation of how SHAP values were calculated and interpreted would enhance understanding.**

Thank you for your valuable comment. In response to your suggestion, we have provided a clearer explanation of how SHAP values were calculated and interpreted. The calculation of SHAP is described in line 249. We utilized the SHAP package in Python, where the SHAP value for a given feature quantifies its impact by comparing the prediction accuracy of a model excluding that feature with the prediction accuracy of a model including it. The detailed calculation method is documented in the SHAP package manual (https://shap.readthedocs.io/en/latest/api.html), and we have incorporated this information into the manuscript to enhance clarity.

5. **The beesworn diagram of the SHAP was not provided.**

Thank you for your valuable comment. In response to your suggestion,the beeswarm diagram is provided in Supplementary Figure S2. In this diagram, red plots indicate high feature values, and a large collection of red dots on the right side suggests that higher feature values increase the likelihood of classification into that category. We have included an explanation and interpretation of this in line 254 of the manuscript.

6. **Why might static features not improve model performance as expected?**

Thank you for your valuable comment. A possible explanation is that the most influential predictive variables were primarily associated with laboratory test results, while the contribution of vital sign variables modeled as time-series data was minimal. We have incorporated this discussion into the Discussion section to clarify this point.

7. **What are the future recommendations and implications of this research?**

Thank you for your insightful comment. Integrating findings from previous studies with our results suggests that developing a predictive model using random forest or recurrent neural networks, incorporating three key factors—static variables influencing aPTT such as coagulation factors and inflammatory markers, time-series data related to severity scores such as SOFA and APACHE scores, and precise information on cumulative heparin dosage—could enhance the model's generalizability and clinical applicability. We have added this discussion to the Discussion section to address future recommendations and implications of our research.

8. **We don’t know if prediction using ordinary statistical tests could perform similarly or better than ML**

Thank you for your insightful comment. We acknowledge your point, and we agree with your concern. In this study, we did not directly compare ML models with traditional statistical methods, such as logistic regression or generalized linear models. However, ML models are generally more effective at handling complex nonlinear relationships. Given that our study incorporated time-series data of vital signs, resulting in a more complex predictive model, we opted to use machine learning approaches to better capture these dynamic interactions. We have added this discussion to the Limitation section to address this point.

**Reviewer #2:**

1. **There is no clear motivation for considering multiple-infusion settings, nor for distinguishing between the first-infusion and the multiple-infusion settings. All cited references restrict themselves to first infusions, so the added value of considering multiple infusions should be clearly stated. Furthermore, I don't understand why first infusions and multiple infusions are treated separately. Both settings share the same set of features and the same labelling policy, so there is no "technical" reason for a separate treatment.**

Thank you for your insightful comment. We acknowledge the need to clearly articulate the rationale for considering multiple-infusion settings and distinguishing them from first-infusion settings.

Previous studies have primarily focused on predicting aPTT responses after the initial heparin administration. However, in clinical practice, UFH management extends beyond the first dose, requiring multiple administrations and continuous dose adjustments to maintain therapeutic anticoagulation. Despite this, no prior studies have validated ML models for predicting aPTT responses after multiple heparin infusions. Predicting aPTT following multiple administrations is crucial as it more accurately reflects real-world anticoagulation management and accounts for the cumulative effects of heparin, which have been highlighted as a challenge in previous research.

Although both settings share the same set of features and labeling policy, we observed differences in the aPTT distribution between first and multiple infusions, suggesting potential variations in heparin response dynamics. An alternative approach could involve developing a single model that integrates both settings by introducing an additional feature indicating the number of prior infusions a patient has received. However, this approach would increase model complexity, potentially affecting both interpretability and performance. Therefore, we chose to analyze first and multiple infusions separately to better evaluate their respective predictive factors and develop a model that is more clinically applicable.
To clarify this point and better convey the significance of considering multiple infusions, we have revised the Introduction accordingly and made partial modifications to the Discussion section.

2. **In the manuscript, the authors merely mention that the aPTT distribution differs between the two settings (Figure 3), but I wonder if there is a clinical motivation as well. Besides, the authors could just as well have trained a single model for both settings, with an additional feature that indicates how many infusions have been administered to the patient before; this would have had the benefit of a larger data basis to train on.**

Thank you for your valuable comment. Figure 3 illustrates the distribution of aPTT values after both initial and multiple heparin infusions, showing that the first-infusion group exhibits a more imbalanced distribution of treatment classes compared to the multiple-infusion group. Clinically, this variability is likely due to individualized dosing decisions by physicians, which may have led to over- or under-dosing of heparin.

We appreciate your insightful suggestion to integrate both settings into a single model by incorporating an additional feature indicating the number of prior infusions. While this is a valid approach, our study focused on evaluating the impact of incorporating time-series data on predictive accuracy. Therefore, we chose the current model structure to specifically assess the effect of dynamic variables on aPTT prediction.

To clarify this point, we have added this explanation to the Results section of the manuscript.

3. **The sample selection process should be described in more detail. I acknowledge that the authors tried to explain this, e.g., in Figure 1, but there are still some open questions: Is it possible that two distinct samples originating from the same patient share the same baseline and/or target aPTT measurements?**

Thank you for your valuable comment. Regarding whether different samples from the same patient share the same baseline and/or target aPTT measurements, we confirm that in the data used for prediction after multiple heparin doses, it was common for the same baseline aPTT and target aPTT to be shared.

4. **What happens if during a continuous heparin infusion the dosage changes? Is this treated as a "new" administration?**

Thank you for your valuable question. We do not treat dosage changes during a continuous heparin infusion as a "new" administration and have excluded such cases from the analysis. These cases are documented in Supplementary Table S2, along with our response to the following comment.

5. **In Figure 2, why does the number of patients in the first-infusion group (N=837) differ from the total number of eligible patients (N=959)? I'd assume that every eligible patient in particular has a *first* infusion. Clearly describing the precise sample selection process is of utmost importance, for being able to replicate the experiments on new data. I strongly recommend adding 1-2 concrete examples (possibly in the Supplement) to illustrate the selection process. The examples should in particular also cover corner cases, e.g., where the heparin infusion ends before the target aPTT is measured.**

Thank you for your valuable feedback. As you pointed out, certain aspects of the data selection process were unclear, so we have added supplementary materials to clarify these points.

This is because some eligible data that can be used for constructing the multiple-infusion model are excluded from the first-infusion model. Conversely, some data included in the first-infusion model are not part of the multiple-infusion model. Specifically:

294 data points included only in the first heparin infusion model correspond to cases where heparin administration was discontinued. 112 data points included only in the multiple heparin infusion model correspond to cases where the target aPTT was not measured.

To make this distinction clearer, we have added Supplementary Figure S1 to visually represent the relationship between the datasets. Additionally, we have provided detailed exclusion examples in Supplementary Table S2, along with specific case descriptions, to further clarify the selection process. These details have also been reflected in Figure 1.

We appreciate your insightful comments, which have helped us improve the clarity and completeness of our manuscript.

6. **The modelling process is not clearly described. For instance, did the authors perform any kind of hyperparameter tuning? If yes, which hyperparameters were tuned, and what were the possible values? If no, why not? Was the architecture of the "multineural network" (which I suppose is meant to be a multilayer perceptron, MLP) the same as in [4], where it outperforms all other models by a large margin? Why did the authors not consider a recurrent neural network, as in [18], in their experiments with time-series input?**

Thank you for your insightful comments.

In response to your suggestions, we have made the following revisions:

We have added information on the parameters used to build the models with SVC, XGB, Random Forest, and LightGBM (excluding the multineural network) in line 233 and listed them in Supplementary Table S3.For the multineural network, we have also implemented a recurrent neural network (RNN) when dealing with time-series information. Additionally, we conducted hyperparameter tuning using a grid search to optimize model performance. These modifications have been incorporated into line 221 and are described in Supplementary Table S3.

We appreciate your valuable feedback, which has helped improve the clarity and completeness of our manuscript.

7. **There is no comparison to a simple baseline, e.g., logistic regression on the top-1 or top-3 features revealed by SHAP. When doing ML experiments, it is important to justify the added value of complex ML models. In this particular case, one might assume that knowing the baseline aPTT alone might already be sufficient to predict the target aPTT with reasonable accuracy; Figure 4 seems to confirm this. (In a related case, there is an ongoing discussion about the added value of the complex, proprietary Hypotension Prediction Index vs. the current mean arterial pressure [MAP] value for predicting future MAP values. So, it would be interesting to know what happens if baseline aPTT, and maybe BHC after SA, were used as the sole predictor variables.**

Thank you for your valuable comments. We fully acknowledge your point, and we agree that this possibility is worth considering. However, ML models are generally more effective at handling complex nonlinear relationships. Given that our study incorporated time-series data of vital signs, resulting in a more complex predictive model, we opted to use machine learning approaches to better capture these dynamic interactions. To address this concern, we have added this discussion to the Limitation section. We appreciate your insightful feedback, which has helped improve the clarity and completeness of our manuscript.

8. **There is no external validation of the proposed approach. This is particularly disappointing, as the authors analyzed data from 6 different hospitals, and could therefore have employed a leave-one-hospital-out cross-validation policy, for instance. Alternatively, any of the MIMICs could have been used for external validation, too, like in related studies [3, 4, 5].**

Thank you for your valuable feedback. We understand your concern; however, patient characteristics varied across the six hospitals, and differences in data quantity and patient populations made it challenging to clearly separate them due to sample size limitations. To enhance clarity on this point, we have added Supplementary Table S5.

Regarding the use of other external databases, we also considered utilizing MIMIC. However, verifying the correctness of drug administration start and end times, as well as performing the necessary data cleaning, would require a significant amount of manual effort. Therefore, we decided not to use it in this study and consider it as a topic for future research.

9. **Lines 82-83, "Few studies have examined aPTT changes across multiple heparin doses.": I quickly scanned the cited literature and did not find any such study. Either explicitly refer to one such study here, or rephrase the sentence if there is none.**

Thank you for your feedback. Based on the reviewer's suggestion, we have revised the Introduction accordingly.

10. **Line 133: The rationale for chosing 6-24 hours after heparin administration as the "target interval" should be briefly explained, in particular since related studies use different intervals.**

Thank you for your valuable comment. In Japanese clinical practice, aPTT testing is typically performed approximately 6 hours after the start of heparin administration. Considering this practice and referencing other studies, we adopted a 6–24 hour range to ensure alignment with clinical practice. This information has been added to line 167 for clarification.

11. **Lines 138-139: [3, 4, 5, 18] all set the upper threshold of the normal-therapeutic range to 100 s, so there should be some justification why it is set to 80 s here.**

Thank you for your valuable comment. In clinical practice, the normal therapeutic range is generally set at 1.5 to 2 times the patient's reference value. If ECMO is used, this range is doubled. Since the normal reference range is 40 seconds, we have set ours at 80 seconds, as indicated on line 168.

12. **Lines 145-146: I do not quite understand why the elapsed time between baseline- and target aPTT measurements is included as a predictor variable. In an application setting, this information is not available at prediction time, so including it as a predictor variable seems strange at first glance. Furthermore, could this introduce hidden bias, e.g., if aPTT is measured only under certain circumstances? Is there a statistical correlation between the elapsed time and the target aPTT value? I understand that all related studies include this variable as well, so it seems to be common practice. But a short explanation and justification would be helpful.**

Thank you for your valuable comments. First, we would like to explain why we included the elapsed time between aPTT measurements as a predictor variable. In clinical practice, when administering medication, the dosage and infusion rate are determined while considering the scheduled aPTT test to be performed several hours later. Therefore, since the timing of the test is already anticipated, we do not consider it problematic to include it as a variable. However, we acknowledge that the variation in testing time may be influenced by the patient’s condition, which could introduce bias. To address this concern, we have included this point as a limitation in line 419. Regarding correlation, since the amount of heparin administered has a significant impact on aPTT, we assume that elapsed time related to medication dosage is also an important factor. However, we have not confirmed a statistical correlation in this study.

13. **Line 173: Were variables standardized before or after k-NN imputation?**

Thank you for your insightful comments. Variable standardization was performed before 5-NN imputation. In response to the reviewer's suggestion, we have added a note on line 207 to clarify this point.

14. **Lines 196-197: Does this apply to the results presented in the main text (Table 2), or only to Table S3 in the Supplement? If the former, was it over- or undersampling, and what's the purpose of Table S3 then? If the latter, this statement is misleading and should be rephrased. In either case, were the test sets resampled, too, or only the training sets?**

Thank you for your valuable comment. We intended the latter approach and, following the reviewer's suggestion, applied it only to Table S3 in the Supplement (now renamed Supplemental Table S10). The purpose of resampling was to evaluate whether applying it to the Random Forest model, which had the highest accuracy, could further improve its performance. Resampling was performed only on the training data and was not applied to the test data. This was done to assess whether the model could still classify imbalanced test data effectively.

15. **Line 201: I do not know the term "multineural network", and a quick internet search did not produce any meaningful results either. Do you mean multilayer neural network, or multilayer perceptron?**

As you correctly assumed, we are referring to a multilayer neural network. To enhance clarity, we have added an explanation on line 233. Thank you for your valuable comment.

16. **Lines 219-220: Why compare the first-infusion group to the multiple-infusion group? It would be more interesting to compare the three classes.**

Thank you for your valuable comment. We categorized the data into these two groups because we considered that the distribution might differ between the first-infusion and multiple-infusion groups. Additionally, the grouping was determined based on the sample size considerations.

17. **Figure 3: There are clearly visible spikes at 100s and 180s. Do you have any explanations for them? Could these be data artifacts, e.g., censored values like ">100s" and ">180s"? If yes, a short statement would be helpful.**

Thank you for your valuable comment. We have confirmed that some facilities have such a censored value. To enhance clarity, we have indicated this on line 300.

18. **Line 249: "strong ability to differentiate" is a strong exaggeration, in my opinion: ROC-AUC values between 0.7 and 0.9 are usually regarded as indicating moderate accuracy, see, e.g., https://doi.org/10.1007/s00134-003-1761-8. Please rephrase the sentence.**

Thank you for your valuable comment. Following the reviewer's suggestion, we have revised "strong ability to differentiate" to "moderate ability" (line 308).

19. **Lines 256, 272: What is a (positive) case in a ternary classification problem?**

Prediction accuracy is presented as the average of the accuracy calculated for each of the three categories, also known as macro accuracy. A positive case refers to an instance correctly classified into one of the three classes. The accuracy for each category is provided in Supplementary Table S9.

20. **Lines 294-296: What about [18]? Or does the sentence refer to the previous sentence about multiple heparin infusions? If so, please rephrase.**

Thank you for your valuable suggestion. To avoid any misunderstanding, we have revised the wording accordingly (line 352).

21. **Lines 320-325: I do not quite agree with the conclusions drawn by the authors. First, the benefit of additional biomarkers is questionable, as evidenced by [4]'s excellent results and Figure 4. Second, I regard the absence "complicated" predictor variables a strength of [4]'s model, not a weakness. It actually renders their model *more* clinically applicable in my opinion.**

Thank you for your valuable insights. We acknowledge your perspective and agree with your reasoning. Accordingly, we have removed the relevant discussion from the manuscript.

22. **Table 1: The class distribution is missing and must be added. Also, this and all other tables are difficult to read => add row separators.**

Thank you for your valuable comment. We have added the class distribution to Table 1 as requested. Additionally, to improve readability, we have added row separators to this and all other tables. Furthermore, we have included detailed patient information by class for baseline and target aPTT in Supplementary Tables S6 and S7.

23. **Table 2: Report class-wise performance metrics as well, at least in the Supplement. Macro-averages alone are insufficient to get a feeling of the clinical usefulness of the models, in partiular since not all misclassifications might be equally "bad" from a clinical perspective.**

Thank you for your valuable comment. We acknowledge the importance of reporting class-wise performance metrics to better assess the clinical usefulness of the models. As requested, we have included performance indicators for each class in Supplementary Table S9.

**Reviewer #3:**

1. **Since there is no dedicated section for previous work, the introduction should cover insights about the gap and what others have done in this domain. The introduction is very short and lacks the depth of analysis on published research. You need to discuss how is this study different from others.**

Thank you for your insightful comment. We agree with your suggestion and have revised the Introduction to provide a more comprehensive discussion of previous research and clearly highlight the gap addressed by our study.

2. **Sentences line 80 and 82 require referencing**

Thank you for your valuable comment. We have added references to the relevant sections to clarify the sources of information and ensure proper citation.

3. **Line 86: mention the dataset size explicitly. Line 87: spell out the static and dynamic features**

Thank you for your valuable comment. Based on feedback from other reviewers, we have made substantial revisions to the structure of the Introduction. Additionally, we have spelled out "static and dynamic features" for clarity.

4. **In Data Source can you add a table of dataset details from the 7 hospitals (show stats of your dataset)**

Thank you for your valuable comment. In response to the reviewer's suggestion, we have added a table detailing the dataset from each of the seven hospitals in Supplementary Table S5.

5. **In line 124, is there a time frame for when aPTT is conducted after the bolus dose to be included? (Noticed it is mentioned in line 190, it is better to mention this in inclusion/exclusion criteria)**

Thank you for your valuable comment. In response to the reviewer's suggestion, we have revised the manuscript to include the time frame for aPTT measurement after the bolus dose in the inclusion/exclusion criteria. Additionally, examples of exclusions are provided in Supplementary Table S2.

6. **In line 138 you defined sub-therapeutic (< 40 s), normal-therapeutic (40–80 s), and supra therapeutic (> 80 s), is this consistent with other research ?? I noticed therapeutic range is defined as 70-100 in other work Can you justify this selection?**

Thank you for your valuable comment. In clinical practice, the normal therapeutic range is generally set at 1.5 to 2 times the patient’s reference value, and if ECMO is used, it is doubled. Since the normal reference range is 40 seconds, we set our upper threshold at 80 seconds, as indicated on line 168. While some studies define the therapeutic range as 70–100 seconds, our selection was based on standard clinical practice and the commonly used reference values in ICU settings.

7. **Since this is a multiclassification (3 categories) it is not clear to me how you calculated performance metrics (is it micro-precision or macro-precision?) can you provide precision and recall for every category (sub-therapeutic, normal-therapeutic, and supra-therapeutic).**

Thank you for your valuable comment. The F1 score, precision, recall, ROC-AUC, and PR-AUC for prediction accuracy are reported as macro accuracy, which is calculated as the average of the accuracy across the three categories (sub-therapeutic, normal-therapeutic, and supra-therapeutic). Additionally, precision and recall for each category are provided in Supplementary Table S9. A detailed explanation of the macro accuracy calculation method is included in Supplementary Table S4.
